# Prospects and limits of marker imputation in quantitative genetic studies in European elite wheat (*Triticum aestivum* L.)

**DOI:** 10.1186/s12864-015-1366-y

**Published:** 2015-03-11

**Authors:** Sang He, Yusheng Zhao, M Florian Mette, Reiner Bothe, Erhard Ebmeyer, Timothy F Sharbel, Jochen C Reif, Yong Jiang

**Affiliations:** Department of Breeding Research, Leibniz Institute of Plant Genetics and Crop Plant Research (IPK) Gatersleben, D-06466 Stadt Seeland, Germany; KWS LOCHOW GMBH, D-29296 Bergen, Germany

**Keywords:** Elite wheat, Map-dependent imputation, Map-independent imputation, Intensive simulation, genomic selection, Association mapping

## Abstract

**Background:**

The main goal of our study was to investigate the implementation, prospects, and limits of marker imputation for quantitative genetic studies contrasting map-independent and map-dependent algorithms. We used a diversity panel consisting of 372 European elite wheat (*Triticum aestivum* L.) varieties, which had been genotyped with SNP arrays, and performed intensive simulation studies.

**Results:**

Our results clearly showed that imputation accuracy was substantially higher for map-dependent compared to map-independent methods. The accuracy of marker imputation depended strongly on the linkage disequilibrium between the markers in the reference panel and the markers to be imputed. For the decay of linkage disequilibrium present in European wheat, we concluded that around 45,000 markers are needed for low cost, low-density marker profiling. This will facilitate high imputation accuracy, also for rare alleles. Genomic selection and diversity studies profited only marginally from imputing missing values. In contrast, the power of association mapping increased substantially when missing values were imputed.

**Conclusions:**

Imputing missing values is especially of interest for an economic implementation of association mapping in breeding populations.

**Electronic supplementary material:**

The online version of this article (doi:10.1186/s12864-015-1366-y) contains supplementary material, which is available to authorized users.

## Background

Imputing missing values is crucial for molecular marker data sets generated by methods with inherent high levels of missing data, for example genotyping-by-sequencing (GBS) [1]. This also holds for approaches aiming to reduce genotyping expenses by combining high-density marker profiling of a population subsample with medium-density marker profiling for the majority of population members. Accurate imputation is important for maximizing the power of detecting causal polymorphisms underlying complex traits [2,3].

Imputation algorithms can be classified into map-dependent and map-independent algorithms. Map-dependent methods impute missing values utilizing available linkage information [2]. In contrast, map-independent algorithms do not use the linear order of markers [1,4]. The accuracy of missing value imputation is expected to be lower for map-independent algorithms in comparison to map-dependent ones which exploit additional information. The magnitude of such differences in accuracy, however, is not known. Despite their lower expected accuracy, map-independent algorithms are relevant for species for which dense and high-quality genetic or physical maps are absent.

Several factors can influence the accuracy of imputing missing values for particular markers [5]. Increasing reference population size enhances imputation accuracy [6-8]. The accuracy of imputation benefits also if genotypic information is available for markers tightly linked to those being imputed [5,8]. In addition, allele frequency impacts the imputation of missing sites, with lower expected imputation accuracy for rare variants [5,8]. The interplay between population size, allele frequency, and linkage disequilibrium, however, has not yet been examined in detail despite the potential interactions of these factors.

Imputed molecular marker data are often used for population genetic and quantitative genetic studies [1,3,4]. The impact of imputation accuracy on diversity analyses has been investigated by analyzing GBS data from rice, maize, and wheat with a map-independent algorithm [4]. The findings revealed that estimating heterozygosity, inbreeding coefficients, and genetic differentiation were substantially biased when missing values were imputed. The effect of imputing missing values in GBS data for the purpose of genomic selection has been examined empirically in maize, wheat, and barley [1]. The prediction accuracy of genomic selection increased if missing values were imputed in contrast to a scenario excluding the missing marker data. The power to detect quantitative trait loci (QTL) increased in genome-wide association mapping studies in human and animal populations if missing values were imputed [9,10]. Analyzing the linkage disequilibrium between true and imputed SNPs in barley additionally supported the idea that QTL detection may be improved by marker imputation in plant populations [5]. Nevertheless, in-depth studies determining the impact of imputing missing values on the power of genome-wide association mapping in plants are still lacking.

Here, we draw upon published data derived from a diversity panel consisting of 372 European wheat varieties [11]. All lines had been genotyped with 9 k [12] and 90 k SNP arrays [13], which allowed us to simulate low to high marker density and GBS-like imputation scenarios. The main goal of our study was to investigate the implementation, prospects, and limits of marker data imputation for quantitative genetic studies. In particular, we (1) contrasted the imputation accuracy of one map-independent and three map-dependent algorithms under varying reference population sizes, (2) studied the influence of linkage disequilibrium and minor allele frequency on imputation accuracy, and (3) investigated the effect of imputation on the precision of diversity studies, the accuracy of genomic selection, and on the power of QTL detection in genome-wide association mapping.

## Methods

### Datasets

We used genotypic information based on previously published 90 k SNP array data from 372 European wheat varieties [14]. In addition, we used information on marker positions of SNPs which were present on a previously published 9 k SNP array [14]. Two of the included lines did not differ with respect to their 9 k SNP array profile. Hence, one of them, variety *Exotic* according to [14] was excluded from analysis. After performing quality checks to exclude those markers that were monomorphic and for which genetic map information was unavailable [15], 9,926 SNPs remained for the 90 k SNP array and 1,573 SNPs remained for the 9 k SNP array (from here on referred to as the original 90 k SNP and original 9 k SNP marker data sets, respectively).

### Imputation scenarios

We randomly divided the 371 individual lines into a reference and a test population in order to evaluate the effects of low to high marker density imputation. We assumed that the reference population was fingerprinted with the 90 k SNP array and the test population only with the 9 k SNP array (Additional file 1: Figure S1). Marker data for the 8,353 SNPs present in the 90 k but not in the 9 k SNP marker data sets were treated as missing values for lines from the test population, and represented the targets for imputation. We assumed different reference population sizes of 50, 100, 200, and 300 out of 371 lines. The sampling for each reference population size was repeated 10 times in order to reduce random error [1,8]. Data sets generated in this way are referred to as low to high marker density data in the following.

We also implemented imputation on randomly excluded empirical data for mimicking GBS-derived marker data. For each line within the population of 371 lines, the 90 k SNP array marker data were randomly masked with the four missing value levels of 72.8%, 61.5%, 38.8%, and 16.1%. These four levels correspond to the missing data rates for the four scales of low to high marker density imputation with 50, 100, 200, and 300 out of 371 lines in the reference population (Additional file 1: Figure S1). The resulting randomly depleted data sets are here forth referred to as GBS-like marker data.

### Imputation approaches

We used three map-dependent imputation algorithms which have been widely used in animal and plant genetics (Beagle v3.3.2 [16], FImpute v2.2 [17], and IMPUTE2 v2.3.0 [18]). In addition, we applied one map-independent algorithm (Random Forest regression). Random Forest regression had performed best among 5 map-independent methods according to a recent study [1].

The Beagle algorithm [16] exploits hidden Markov models (HMM) to infer haplotypes of individual lines and to impute missing values. First, data completion is initialized by imputing the missing values based upon allele frequencies with random phasing of the haplotypes of the individual lines. The initial data set is then used to build localized haplotype-cluster models, which represent a special class of HMM. Each model proceeds along a chromosome and has the same number of levels as the number of markers. At each level, the hidden states are the clusters of haplotypes [19]. The emitted symbols are the alleles. A forward-backward algorithm [20] is applied to estimate the probabilities of each possible haplotype based on the genotype information. Then, new haplotypes for the individuals are sampled according to the conditional probabilities as input for the next iteration. The procedure is repeated until the final iteration, where the Viterbi algorithm [20] is applied to infer the most-likely haplotypes for all individuals. Thus, the missing data points are imputed simultaneously in this step.

The FImpute population-based algorithm [17] is based on a haplotype-matching process which assumes that all individuals in the population are related. Sliding windows are used to search for consistent haplotype segments assuming that each imputed individual has a recent common ancestor within the reference population. The initial window size is large and moves along each chromosome in steps with a certain overlap. Then, the window size is steadily reduced as the procedure is repeated. Finally, the most likely haplotype is determined for each individual line based on the frequency of haplotypes in the reference population. Moreover, the number of hits from the window analysis is computed and the missing data points are imputed.

The IMPUTE2 algorithm [18] is an enhanced version of the IMPUTE1 algorithm [21] and is based on HMM and Markov chain Monte Carlo (MCMC) iterations. It differs from Beagle mainly in two points. First, it separates the procedures of inferring haplotypes and imputing missing genotypes. Second, it divides the data into (1) markers with information present in both the reference and the test set (the T part), and (2) markers with information present only in the reference set (“untyped”, the U part). Beagle in contrast builds a joint model for all individual lines at all loci. The algorithm IMPUTE2 starts by guessing the haplotypes in the T part and then performs a number of MCMC iterations. The first step in each iteration involves the loci of the T part. For each individual line, new haplotype pairs are sampled based on the probability derived from the genotype and the currently estimated haplotypes of all other individual lines, in addition to a scale parameter. In the second step, missing alleles in the U part are then imputed based on the probability derived from the results of the calculation in the first step.

The Random Forest algorithm is an advanced machine learning approach [22,23]. Molecular markers are first sorted from the lowest to highest missing data levels. Then, the missing values are initialized through a simple imputation method (e.g. sampling based on allele frequencies) and the Random Forest regression model is fitted and iterated. For each marker vector y containing missing values, 100 regression trees are grown using the non-missing values *via* bootstrapping. At each node in each tree, a random sample of $\sqrt{n-1}$ predictors, that is, other markers at the same row of the missing part of y, is used as splitting variables, where *n* refers to the number of markers. The terminal node of each tree gives a prediction of the missing part of y. Then, the means of predictions obtained in all regression trees are taken as the imputed values. The above steps are repeated until convergence or 10 times at maximum.

### Imputation accuracy

We used the correlation (cor) between true and imputed marker profiles as the parameter to estimate imputation accuracy. This metric is recommended because it efficiently measures the imputation accuracy for rare variation [8] and is related to the power of detection of genome-wide association scans [24]. Missing data points in the original data set, which are not caused by masking, were excluded from the evaluation.

### Factors affecting the imputation accuracy

We used the r^2^ statistic [25] as a measure of linkage disequilibrium (LD) and examined the association between imputation accuracy and maximum LD between the imputed and observed markers. The values of r^2^ were estimated as r^2^ = (p(AB) − p(A)p(B))/(p(A)p(a)p(B)p(b)), where p(AB) is the frequency of haplotype AB and p(A), p(a), p(B), p(b) is the allele frequencies of two bi-allele loci. Furthermore, we studied the association between minor allele frequency (MAF) and imputation accuracy.

### Effect of imputation on the estimation of Rogers’ distance

We studied the effects of imputation on estimating Rogers’ distances among pairs of genotypes (RD, [26,27]). RD was used as genetic distance measure because it is linearly related to the coefficient of co-ancestry for homozygous lines [28,29] and was estimated as

$$ RD=\frac{1}{m}{\displaystyle \sum_{i=1}^m}\sqrt{\frac{1}{2}{\displaystyle \sum_{j=1}^{n_i}}{\left({p}_{ij}-{q}_{ij}\right)}^2}, $$

where *p*_*ij*_ and *q*_*ij*_ are allele frequencies of the *j* th allele at the *i* th locus in the two individuals under consideration, *n*_*i*_ is the number of alleles at the *i* th locus, and *m* refers to the number of loci. For the low to high marker density imputation scenario, we first calculated the correlation between the RD matrices estimated by the original 90 k and original 9 k SNP marker data as a benchmark. We then estimated the correlations between RD matrices based on different imputed low to high density marker data sets and the original 90 k SNP marker data. Here, the comparison was exclusively based on imputed markers in data for the lines from the test population in order to focus solely on the accuracy of imputed marker values. Significance of the correlations between RD matrices was tested according to a Mantel test [30].

Since there exist no markers without missing values in the GBS-like marker data sets generated by random depletion, we instead first estimated RD matrices among pairs of genotypes based on the genotypic profiles by omitting for each pair of lines markers with missing values. We then calculated the correlation between non-imputed RD matrices and the RD matrices estimated using the original 90 k SNP marker data. We subsequently estimated the correlations between RD matrices based on different imputed GBS-like marker data sets and the original 90 k SNP marker data. Here, the comparison was based on individual lines from the total population comprising 371 genotypes.

### Effect of imputation on genomic selection

We performed simulations to study the effect of imputation on the prediction accuracy of genomic selection. Heritability was assumed to be 0.91. Every marker was set to contribute equally (i.e., $\frac{1}{9926}$) to the total genetic variance in order to mimic a genetic architecture typical for complex traits such as grain yield. To investigate the effects of imputation accuracy on genomic selection, we calculated accuracies of prediction using imputed low to high marker density data sets derived from the four different imputation algorithms, four different reference population scales, and ten technical replications. For low to high marker density imputation, the results were then compared with the accuracies of prediction by ridge regression best linear unbiased prediction (RR-BLUP) [31] using the original 9 k and original 90 k SNP data sets. For the GBS-like scenario, we estimated the genomic relatedness matrix of the total population based on the randomly depleted 90 k SNP data sets without imputation, and then used the genomic best linear unbiased prediction (GBLUP) model to predict genotypic values as outlined in detail elsewhere [32]. The accuracies of prediction of GBLUP were compared to those calculated based on the different imputed marker data panels. It is important to note that GBLUP and RR-BLUP are equivalent [33] but GBLUP facilitates a more proper handling of missing data for the GBS-like scenario.

### Effect of imputation on genome-wide association scans

We further performed a simulation study to investigate the effect of imputation on genome-wide association mapping. Heritability was set again to 0.91. Simulation was performed assuming one QTL explaining 10% of the genetic variance for which a perfect marker was available in the 90 k, but not in the 9 k SNP array. The remaining 9,925 markers were assumed to contribute equally (i.e., $\frac{1}{9925}$) to the remaining genetic variance. The MAF of the QTL was chosen to be larger than 0.3.

For low to high marker density imputation, we selected SNPs with different levels of LD (around 0.1, 0.5 and 0.9) between the QTL and the most closely linked markers in the 9 k SNP data set. We compared the detection frequencies of QTLs in the following 3 scenarios: (1) in the total population fingerprinted with the original 90 k SNP marker data, (2) in the reference population fingerprinted with the original 90 k SNP array, and (3) in the total panel of 371 lines for which non-available 90 k SNP marker data had been imputed. In addition, the detection frequencies of the most closely linked SNP markers present in the 9 k data set from the total population was included in the comparison.

For the GBS-like imputation scenario, also three levels of LD were considered. Here, LD was measured between the QTL and the most closely linked markers in the original 90 k SNP data set. We compared the detection frequencies of the QTL in the following 3 scenarios: (1) in the total population fingerprinted with the original 90 k SNP marker data set, (2) in the assumed available genotypic profiles of the total population of 371 lines irrespective of imputation, and (3) in the total panel for which non-available 90 k SNP array data had been imputed by three imputation approaches.

Genome-wide association mapping was performed based on a linear mixed model approach [34]. The model can be described as *y* = *μ* + *αm* + *Xg* + *e*, where *y* is the vector of simulated trait values for each genotype, *μ* is the vector of common intercept terms, *m* is the effect of the marker being tested, *α* denotes the vector of scores of the marker, *g* is the vector of genotype effects with the corresponding design matrix *X* and *e* is a residual term. The marker effect was assumed to be fixed, while all other effects were assumed to be random. Correction for population stratification was done by assuming $g \sim N\left(0,2K{\sigma}_G^2\right)$, where *K* is a kinship matrix estimated as 1 minus the RD and *σ*_*G*_^2^ is the genotypic variance estimated by a maximum likelihood (REML) approach. Significance of marker-trait associations was tested based on the Wald-F statistic.

## Results

### Accuracy of imputing missing values with map-dependent and independent algorithms

The accuracy of imputing missing values was quantified for different algorithms by estimating the correlation between the original high density 90 k SNP marker profiles and the imputed marker profiles (Additional file 1: Figure S1). In this scenario, the markers for which missing values were imputed were the ones included in the 90 k SNP but not in the 9 k SNP array. The map-dependent methods Beagle, FImpute, and IMPUTE2 led to higher imputation accuracies than the map-independent method Random Forest (Table 1). Imputation accuracy benefited from increasing the size of the reference population. Nevertheless, even for the smallest reference population size of 50 out of 371, the average correlation between true and imputed marker profiles amounted up to 0.74 for IMPUTE2.

**Table 1:** **Accuracies of imputing measured as average correlations (cor) between observed and estimated marker genotypes**

**Algorithm**	Ref 50*	Ref 100*	Ref 200*	Ref 300*
	**cor**	**cor**	**cor**	**cor**
Low to high marker density				
Beagle	0.61	0.70	0.75	0.78
FImpute	0.68	0.73	0.77	0.80
IMPUTE2	0.74	0.77	0.81	0.84
Random Forest	0.56	0.61	0.66	0.69
Genotyping-by-sequencing-like				
Beagle	0.76	0.85	0.92	0.95
FImpute	0.59	0.79	0.91	0.95
IMPUTE2	0.68	0.82	0.91	0.95
Random Forest	0.54	0.64	0.75	0.83

Map- dependent (Beagle, FImpute, and IMPUTE2) and map-independent (Random Forest) algorithms were applied with reference population sizes of 50, 100, 200, and 300 lines out of 371, and imputing was performed for a low to high marker density and for a GBS-like data scenario.

*For GBS-like imputation scenarios, Ref 50, Ref 100, Ref 200, and Ref 300 refer to missing value rates 72.8%; 61.5%; 38.8%; 16.1% for all lines of the population, corresponding to scenarios with reference population sizes of 50, 100, 200, and 300, of the total of 371 lines.

We contrasted the above scenario with a scenario mimicking imputation of missing values for genotyping by sequencing (GBS)-like data sets (Table 1). Estimated values of correlations between true and imputed marker profiles for GBS-like data were consistently higher than for imputing from low to high density marker profiles. The only exceptions were results obtained with the algorithms FImpute, IMPUTE2 and Random Forest for the smallest reference population size of 50 out of 371. Interestingly, the accuracy of imputing missing values benefited from an increase in the reference population size more for the GBS-like than the low to high imputation scenario (Table 1). Furthermore, we observed different rankings among algorithms comparing the two imputation scenarios.

### Imputation accuracy is influenced by linkage disequilibrium and minor allele frequency

**Figure 1:**
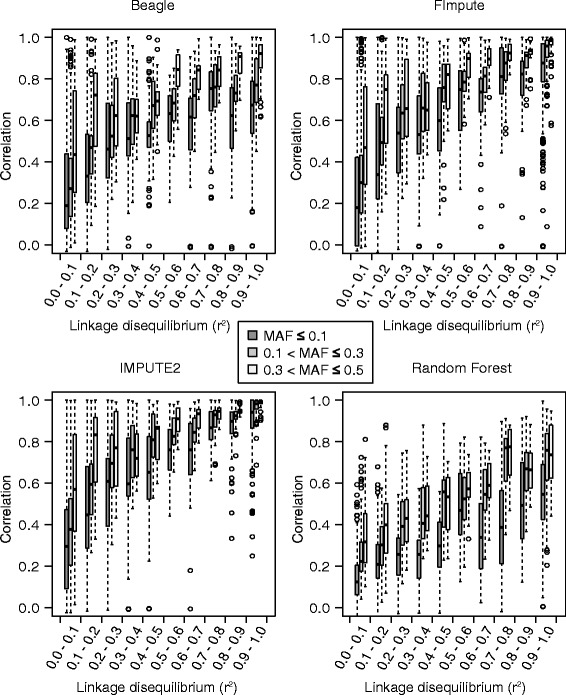
**Linkage disequilibrium influences the accuracy of imputing missing values.** The relationship between linkage disequilibrium (as measured by r^2^ between 90 k SNPs and the respective most closely linked 9 k SNPs) and the average correlation between observed and imputed genotypic data, as calculated using map-dependent (Beagle, FImpute, and IMPUTE2) and map-independent (Random Forest) imputation algorithms, for a reference population size of 50 out of 371 lines. Trends are shown as boxplot displays separately for three minor allele frequency (MAF) classes.

Average LD among adjacent loci in the total population amounted to 0.52 with a standard deviation of 0.39 (Additional file 2: Figure S2). We analyzed the influence of LD and MAF on imputation accuracy with varying reference population sizes. Figure 1 summarizes the results for a reference population size of 50 out of 371 individuals for the low to high density scenario. Similar trends were observed for all other reference population sizes independent from the applied imputation scenario (data not shown).

The impact of LD on the accuracy of imputing missing values was examined separately for three different MAF classes (Figure 1). We focused on LD between SNPs from the 90 k marker data set and those from the 9 k marker data set that were most closely linked to them. The average MAF in the total population amounted to 0.13 with a standard deviation of 0.14. The imputation accuracy improved non-linearly with increasing LD between the 90 k array SNPs and most closely linked 9 k array SNP for all four applied imputation algorithms irrespective of the MAF class. The coefficient of variation for accuracy of imputing missing values decreased substantially with increasing LD for all map-dependent methods. This trend was less pronounced for the map-independent method Random Forest.

We further studied the influence of the MAF on the correlation between true and imputed marker profiles separately for four classes of LD (Additional file 3: Figure S3). Despite higher average correlations between true and imputed marker profiles for higher LD classes, we observed that the accuracy of imputing missing values increased with increasing frequencies of the minor allele irrespectively of the extent of LD. This trend was, however, by far less pronounced than the influence of LD on the accuracy of imputing missing values.

### Effect of imputation accuracy on estimated landscape of relatedness

For the low to high density marker imputation scenario, we used the correlation of all pairwise Rogers’ distances (RD) based on the original 9 k in comparison to the original 90 k SNP array as benchmark. The correlation amounted to 0.95 (Table 2). This value was not reached by imputing missing values with the map-independent algorithm Random Forest. In contrast, the correlation of RD estimated based on imputed and observed marker data was 0.96 for the map-dependent algorithm IMPUTE2 even for the smallest reference population size 50 out of 371 (Table 2).

**Table 2:** **Correlations between Rogers’ distance matrices of the individual lines of the test population**

**Data set**	**Ref 50**	**Ref 100**	**Ref 200**	**Ref 300**
	**cor**	**cor**	**cor**	**cor**
9 k panel	0.95	0.95	0.95	0.95
Beagle	0.83	0.92	0.95	0.96
FImpute	0.95	0.96	0.97	0.97
IMPUTE2	0.96	0.97	0.98	0.98
Random Forest	0.61	0.61	0.61	0.66

Estimates are based solely on imputed parts of data sets (90 k SNP minus 9 k SNP data) and the original 90 k SNP data set, as well as the correlation between Rogers’ distance matrices of the original 9 k and original 90 k SNP data sets. Different imputed low to high marker density data sets were generated by map- dependent (Beagle, FImpute, and IMPUTE2) and map-independent (Random Forest) imputation algorithms for reference populations of 50, 100, 200, and 300 out of 371 lines. All correlations were significantly larger than zero (P < 0.01) according to a Mantel test.

For the GBS-like imputation scenario, the correlation between RD matrices between randomly depleted 90 k SNP marker data without imputation and the original 90 k SNP data set was regarded as benchmark. For imputation with the map-independent method Random Forest, the benchmark was not reached with missing data rates of 72.8% or 61.5% (Additional file 4: Table S1). In contrast, with imputation by either of the three map-dependent methods Beagle, FImpute, and IMPUTE2, the correlation between RD estimates based on imputed and original 90 SNP data was higher than the respective benchmark for all missing data levels.

### Effect of imputation on the accuracy of prediction of genomic selection

**Figure 2:**
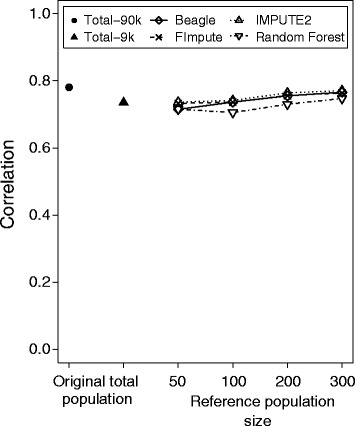
**Imputing from low to high density has a limited effect on the accuracy of genomic selection.** Correlation between results of genomic selection based on true and predicted genotypic values applying genomic selection for the original 90 k (Total-90 k) and 9 k SNP data sets (Total-9 k) for the total 371 lines, as well as for imputing low to high density marker data applying map-dependent (Beagle, FImpute, and IMPUTE2) and map-independent (Random Forest) imputation algorithms, for reference population sizes 50, 100, 200, and 300 out of 371 lines.

To examine the impact of imputing missing marker data on the accuracy of prediction of genomic selection, we simulated a scenario for a complex trait. We used the accuracy of prediction realized with the original 9 k and the original 90 k SNP data sets as points of reference for the low to high density marker scenario. The accuracy of prediction for the total population increased by only 7% (0.73 to 0.78) when using the full 90 k instead of the 9 k SNP marker profiles (Figure 2). The accuracy of prediction of genomic selection based on the original 9 k SNP marker data was outperformed by imputing missing values with the map-independent algorithm Random Forest only when a reference population size of 300 out of 371 individual lines was used. In contrast, the accuracy of prediction of genomic selection based on the data sets imputed with the map-dependent algorithm IMPUTE2 outperformed the accuracy of prediction of genomic selection based on the original 9 k SNP marker data even with a small reference population size of 50 out of 371 individual lines.

**Figure 3:**
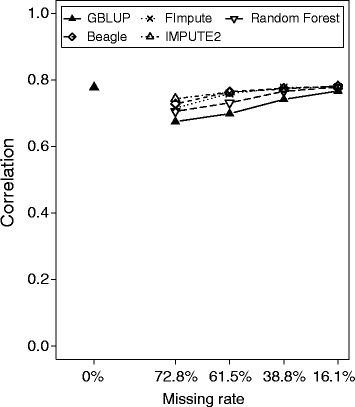
**Imputing improves accuracy of prediction of genomic selection based on GBS-like data sets.** Correlation between true and predicted genotypic values applying genomic selection for the original 90 k SNP data set (GBLUP, 0%), as well as for genotype-by-sequencing-like data sets where missing values were not imputed (GBLUP) or were imputed with map-dependent (Beagle, FImpute and IMPUTE2) and map-independent (Random Forest) algorithms for rates of missing values of 72.8%, 61.5%, 38.8% and 16.1% in the total population of 371 lines.

As a benchmark to evaluate the accuracy of genomic selection based on imputed GBS-like data sets, we used the accuracy of prediction realized with a GBLUP approach based on a kinship matrix estimated with original 90 k SNP marker data or the non-imputed marker data. The accuracy of the GBLUP approach based on the original 90 k SNP marker data amounted to 0.78 (Figure 3). Prediction accuracies of GBS-like data sets profited slightly when missing marker data were imputed (Figure 3). Applying map-dependent as well as map-independent algorithms led to higher accuracies of prediction as compared to the GBLUP across all examined scenarios with missing data rates of 72.8%, 61.5%, 38.8% and 16.1%.

### Effect of imputation accuracy on power of association mapping

**Figure 4:**
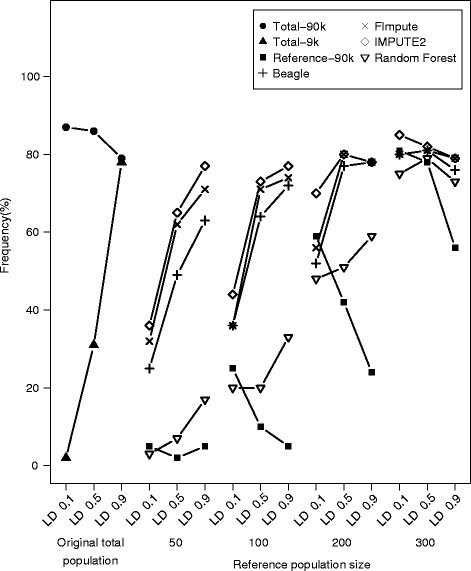
**Imputing from low to high marker density increases the power of association mapping.** Detection frequency of a major QTL explaining 10% of the genotypic variance in the total population based on the 90 k SNP data set (Total-90 k) and the SNP present in the 9 k data set most closely linked to the SNP in the 90 k data set (Total-9 k). Detection frequency of a major QTL in the reference population with sizes from 50 to 300 individuals fingerprinted with the 90 k SNP array (Reference-90 k). Detection frequency of major QTL with varying degrees of linkage disequilibrium (r^2^) between the QTL and closest linked 9 k SNP marker estimated in the total panel for which depleted 90 k SNP array data have been imputed for the test population with map- dependent (Beagle, FImpute, and IMPUTE2) and map-independent (Random Forest) algorithms for the reference population sizes of 50, 100, 200, and 300 out of 371 lines.

Finally, we approached the impact of missing data imputation on the power of association mapping in a simulation study. We assumed the presence of a QTL with a marker allele frequency above 0.3 which explained 10% of the genotypic variation. Such a QTL could be detected in more than 80% of the performed association mapping runs based on the original 90 k SNP marker data for the whole population. Replacing the original 90 k SNP marker data by imputed data starting from 9 k SNP marker profiles led to reduced QTL detection power for almost all tested reference population sizes and LD values (Figure 4). Nevertheless, imputation using the map-dependent methods increased the power of association mapping in comparison to QTL detection based on non-imputed marker profiles. This increase was more pronounced if a SNP with high LD to the QTL was covered within the 9 k SNP marker data. In comparison, imputation using the map-independent Random Forest method increased the power of QTL detection only slightly, except that a marker within the 9 k SNP data set was tightly linked to the QTL. Similar trends as for the low to high marker density imputation scenario were observed for the GBS-like scenario (Additional file 5: Figure S4).

## Discussion

**Figure 5:**
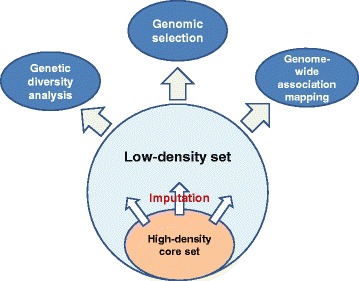
**Strategy for molecular breeding of wheat.** We suggest combining high-density genotyping of a core set of wheat lines with low density and low cost fingerprinting of the entire breeding population followed by imputing of missing marker data for the subsequent quantitative genetic analyses.

In crop improvement programs, molecular markers are employed for (1) studying the relationship among lines [35], (2) QTL mapping in breeding populations [36], (3) marker-assisted foreground- and background selection [37], and (4) genomic selection [38,39]. In this context, the profitability of applying molecular markers strongly depends on the marker density required to achieve the intended purpose and the costs of fingerprinting at this marker density per genotype [40]. One possible strategy to reduce the total costs of such studies encompasses fingerprinting of only a core panel of lines to full depth with a dense marker platform coupled with genotyping of the entire breeding population with less dense and low-cost marker techniques (Figure 5). Low and high density fingerprints are then combined in a second step using imputation algorithms to generate marker data sets for further analysis [2]. The main goal of our study was to investigate the implementation, prospects, and limits of marker imputation for quantitative genetic studies using experimental data collected from a representative sample of European wheat lines [14].

### Imputation accuracy benefits from high-quality physical and genetic maps in wheat

Comparing the performance of different imputation approaches, we observed substantially higher correlations between true and estimated marker profiles for map-dependent than for map-independent imputation methods (Table 1). This can be explained by the use of haplotype information in the map-dependent approaches [2]. Our findings thus clearly stress the importance of the availability of high-quality physical (Beagle algorithm) and genetic maps (IMPUTE2 and FImpute algorithms) for crops in order to implement imputation (Figure 5) in plant breeding. In this light, an ordered draft sequence of the hexaploid bread wheat genome has recently been released [41]. Moreover, considerable effort has been devoted to develop dense consensus maps for wheat [12,13]. Relevant physical and genetic map information is thus becoming available in order to support molecular wheat breeding strategies involving imputation. Hence, we focus our discussion on the results generated for map-dependent methods. For minor crops without detailed map information, map-independent imputation might still seem an option. However, its clear limitations require careful consideration. For instance, the genetic relatedness among genotypes will be biased when diversity is calculated based on genomic data compiled by map-independent imputation (Table 2, Additional file 4: Table S1, [4]).

### Implementation of imputation strategy in wheat

In accordance with previous findings [5,8], we observed that imputation accuracy strongly depended on LD between the markers in the reference panel and the markers to be imputed (Figure 1). In contrast, allele frequency impacted imputation accuracy only marginally if the low density panel contained markers that exhibited at least moderate levels of LD (r^2^ > 0.5) with the imputed markers in the high density panel (Additional file 3: Figure S3). Average r^2^ values of markers with distances of up to 0.5 cM amounted to only 0.32 with a 25% quantile of 0.005 in our panel of European wheat lines (Additional file 2: Figure S2). This fast LD decay in our European elite wheat panel is surprising as LD is expected to decrease substantially slower in wheat as a selfing species [42-45] in comparison to an outcrossing crop such as maize [46-48]. Taking the decay of LD and the length of the genetic map of wheat of around 4,500 cM into account [12], and focusing on the gene space only, one can estimate how many markers will be needed to be determined at minimum. For a scenario involving rare alleles, approximately 45,000 markers would be required in order to facilitate high imputation accuracy.

A previous study combining experimental data of a cattle population with computer simulations [49] suggested that an optimal composition of the reference population could increase imputation accuracy. We examined this issue in our study by inspecting the association between the diversity of the reference population measured as the average RD and the imputation accuracy. For all sizes of the reference population, we observed moderate correlations with an average value of r = 0.4. This indeed hints to the possibility to enhance the imputation accuracy via an optimal composition of the reference population. A more detailed analysis of this prospect in our current study, however, is hindered by the lack of deep genotyping information that would be required to optimize the composition of the reference population [49].

### Influence of missing site structure on imputation accuracy

The distribution of missing marker data points also impacted the accuracy of imputation (Table 1). Random missing data, as applicable for our GBS-like data sets [1], facilitated higher imputation accuracy compared to blocks of missing marker data. The relative advantages or disadvantages of the diverse marker platforms available, however, depend ultimately also on many other factors such as e.g. costs per marker data point, distribution of markers, as well as potential ascertainment bias [50] that cannot be discussed in the framework of this study.

### Association mapping profited most from imputation of missing values

Marker densities only marginally affect the accuracies of genomic selection for complex traits. Typically, a plateau of accuracy is reached with a few hundred markers for bi-parental populations [39,51,52] and with a couple of thousand markers for diversity panels [53,54]. Even slightly lower numbers of markers have been recommended to reliably portray genetic relationships [55]. In accordance with these findings, we observed that accuracies of prediction of genomic selection or relatedness estimation could profit only marginally from imputing missing marker data (Figures 2 and 3; Table 2; Additional file 4: Table S1).

In contrast, association mapping is strongly influenced by the marker density [56], as other than genome-wide prediction it focuses on one QTL at a time [57]. Therefore, missing marker data at a particular locus cannot be compensated by other closely linked loci, which leads to a strong impact on the power of QTL detection [58]. Consistent with this expectation, we observed that the power of association mapping strongly benefited from imputing missing marker data (Figure 4; Additional file 5: Figure S4). Consequently, for association mapping in breeding populations, substantial added value can be generated by complementing routine genotyping performed on an economic marker platform with high density genotyping of a core set of lines for subsequent data imputation. The high density marker data could for instance be generated by re-sequencing, as has been implemented for soybean [59] or maize [60].

In wheat, several relevant agronomic traits such as flowering time [61,62], plant height [63], abiotic stress tolerance [64,65], and disease resistances [66] are expected to be controlled by large effect QTL. Such a genetic architecture with large effect QTLs enables efficient marker-assisted selection. Association mapping can be considered as a promising approach to identify further functional markers in breeding populations [36]. The identified functional markers can be efficiently combined with genome-wide prediction approaches to bridge the gap between marker-assisted and genomic selection [67].

## Conclusions

From the examined quantitative genetic applications, association mapping profited most from imputing missing values. Association mapping is valuable for traits controlled by large effect QTLs, which is the case for a number of the economic important traits in wheat. Consequently, routine implementation of marker imputation provides a powerful tool for marker-assisted wheat improvement.

## Additional files

Additional file 1: Figure S1. Generation of simulated low to high marker density and GBS-like SNP marker data sets. For low to high marker density data simulation, the 371 lines were divided into a reference and a test population, for which availability of 90 k or 9 k SNP marker data sets, respectively, was assumed. Thus, data for SNPs present in the 90 k but not in the 9 k SNP marker data sets were the target for imputation. Sizes of the reference population were set to 50, 100, 200, and 300 out of 371 lines. Further, genotyping-by-sequencing-derived-mimicking data sets were generated by randomly depleting 72.8%, 61.5%, 38.8%, 16.1% of marker information from 90 k data sets for individual lines, thus being comparable to the low to high marker density scenarios involving 50, 100, 200, and 300 reference lines. Additional file 2: Figure S2. Rapid decay of linkage disequilibrium with genetic map distance. Distribution of linkage disequilibrium measured as r^2^ over different genetic map distance classes between SNP marker pairs determined for 371 wheat lines. *No. pairs* represents the amount of SNP pairs within each distance class. Additional file 3: Figure S3. Minor allele frequency impacts the accuracy of imputing missing values. The relationship between minor allele frequencies of 90 k SNPs and average correlations between observed and imputed genotypic data, calculated using map-dependent (Beagle, FImpute, and IMPUTE2) and map-independent (Random Forest) algorithms for a reference population size of 50 individuals. Trends are shown as boxplots for four classes of linkage disequilibrium (LD) levels between 90 k SNPs and the respective most closely linked 9 k SNP. Additional file 4: Table S1. Correlation between Rogers’ distance matrices for imputation in a GBS-like scenario. Additional file 5: Figure S4. Influence of imputing for GBS-like data sets on power of association mapping. Detection frequency of a major QTL explaining 10% of the genotypic variance in the total population fingerprinted with the 90 k SNP array (Total-90 k). Detection frequency of a major QTL in the available genotypic profiles of genotyping-by-sequencing like data sets with missing rates varying from 16.1% to 72.8% (Non-imputed 90 k). Detection frequency of major QTL with varying degree of linkage disequilibrium (r^2^) between the QTL and closest linked SNP marker estimated in the total panel for which missing genotyping-by-sequencing like array data have been imputed with four algorithms (Beagle, FImpute, IMPUTE2 and Random Forest) based on four different missing rates (72.8%, 61.5%, 38.8%, and 16.1%).

## Abbreviations

GBS: Genotyping-by-sequencing
QTL: Quantitative trait locus
SNP: Single nucleotide polymorphism
HMM: Hidden Markov models
MCMC: Markov chain Monte Carlo
Cor: Correlation
LD: Linkage disequilibrium
MAF: Minor allele frequency
RD: Rogers’ distances
RR-BLUP: Ridge regression best linear unbiased prediction
GBLUP: Genomic best linear unbiased prediction
REML: Restricted maximum likelihood
cM: Centimorgan
