# Supplementary material for: Prospects and limits of marker imputation in quantitative genetic studies in European elite wheat (Triticum aestivum L.)
Source: BMC Genomics. 2015 Mar 11;16(1):168. doi: 10.1186/s12864-015-1366-y (PMC4364688; doi:10.1186/s12864-015-1366-y)
Supplement: Additional file 4: Table S1. — Correlation between Rogers’ distance matrices for imputation in a GBS-like scenario. [file 12864_2015_1366_MOESM4_ESM.pdf]

Table S1 Correlation between Rogers' distance matrices for imputation in a GBS-like scenario.

Algorithm	72.8% cor	61.5% cor	38.8% cor	16.1% cor
Non-imputed	0.94	0.97	0.99	1.00
Beagle	0.98	0.99	1.00	1.00
FImpute	0.98	0.99	1.00	1.00
IMPUTE2	0.99	0.99	1.00	1.00
Random Forest	0.89	0.95	0.99	1.00

Estimation was done for the total population of 371 lines based on imputed panels or non-imputed, and the original 90k SNP marker data sets. Different imputed marker data sets were generated using map-based algorithms Beagle, FImpute, and IMPUTE2 as well as map-independent algorithm Random Forest for missing data rates of 72.8%, 61.5%, 38.8%, and 16.1%. All the correlations were significantly larger than zero ($P < 0.01$) according to a Mantel test.
